# Electrochemical Cell-Based Sensor for Detection of Food Hazards

**DOI:** 10.3390/mi12070837

**Published:** 2021-07-18

**Authors:** Jiancheng Zhang, Lixia Lu, Zhenguo Zhang, Liguo Zang

**Affiliations:** Shandong Provincial Key Laboratory of Animal Resistance Biology, Key Laboratory of Food Nutrition and Safety, College of Life Sciences, Shandong Normal University, Jinan 250014, China; zhangjc5785@163.com (J.Z.); 616063@sdnu.edu.cn (L.L.)

**Keywords:** cell, food hazard, electrochemical, biosensor, detection

## Abstract

People’s health has been threatened by several common food hazards. Thus, it is very important to establish rapid and accurate methods to detect food hazards. In recent years, biosensors have inspired developments because of their specificity and sensitivity, short reaction time, low cost, small size and easy operation. Owing to their high precision and non-destructive characteristics, cell-based electrochemical detection methods can reflect the damage of food hazards to organisms better. In this review, the characteristics of electrochemical cell-based biosensors and their applications in the detection of common hazards in food are reviewed. The strategies of cell immobilization and 3D culture on electrodes are discussed. The current limitations and further development prospects of cell-based electrochemical biosensors are also evaluated.

## 1. Introduction

In recent years, people are more and more interested in food safety because of the frequent occurrence of food problems caused by harmful substances around the world. Common hazards in food mainly include biological hazards, chemical hazards and physical hazards [1]. These hazards mainly refer to organisms (especially microorganisms) themselves and their metabolic processes, and the pollution of metabolites (such as toxins) caused by parasites, their eggs and insects to the processing process and products of food materials. Food components (allergens), antibiotics, heavy metals and other hazardous substances in food are also food hazards [2].The incidence rate of foodborne diseases reported by many developed countries has been on the rise recently. Unsafe food containing these common hazards can cause more than 200 diseases. Eating these foods can lead to diarrhea, cancer and even death. According to the data provided by the World Health Organization, about 600 million people around the world get sick every year because of the contaminated foods, and 420,000 die as a result. Two-fifths of the population suffering from foodborne diseases are children, and about 125,000 children die every year. The Centers for Disease Control and Prevention (CDC) estimated that about 48 million people in the United States are infected with foodborne diseases every year, while 128,000 people required hospital treatment and 3000 died from foodborne illness [3]. Diarrhea caused by eating contaminated food is a common disease in food safety incidents, which causes 550 million people to become sick and 230,000 people to die each year. Therefore, it is of great significance to realize the accurate and rapid monitoring of food hazards in the process of food production and consumption [4].

Recently, biosensors have developed rapidly, owing to their high sensitivity, excellent accuracy and fast analysis speed. A biosensor is an instrument that senses a biological substance and converts its concentration into an electrical signal. A biosensor is an analytical tool composed of identification elements including enzymes, antibodies, cells and other biologically sensitive materials, signal amplification devices, and appropriate physical and chemical transducers such as oxygen electrodes, photosensitive tubes, field effect tubes, and piezoelectric crystals [5]. The function of the transducer is to convert various physical, chemical and biological signals into electrical signals, and then, obtain the corresponding results through data analysis and processing. The sensor can produce intermittent or continuous digital signals, and the strength of the signal is proportional to the analytical device.

Biosensors can be divided into cell-based biosensors, molecular biosensors, and tissue biosensors on the basis of their recognition elements [6,7], and cell-based electrochemical sensors have a more outstanding performance. A cell-based electrochemical biosensor is a sort of biosensor that uses cells as sensitive identification elements, which can respond to external stimuli or environmental changes. When cells feel external stimuli, the signal generated by molecular recognition and cell signal transduction go through chemical or physical transducers [8]. The transducer is transformed into a quantifiable and processable electrical signal, which is amplified and output by the secondary instrument. Then, the existence and concentration of the substance to be measured can be known. Cell biosensors based on electrochemical detection can analyze and evaluate cells by measuring current, potential, impedance, conductivity, capacitance and other electrochemical signals, which has become a research hotspot in the field of biosensors [9,10,11,12]. In recent years, with the development of nanotechnology and the enhancement of the interface between sensors and cells, a series of cell-based electrochemical biosensors have been constructed to study cell type, concentration, vitality, proliferation, apoptosis and molecular distribution inside and outside cells. On the other hand, cell-based biosensors can use fixed living cells as sensing elements by stimulating a specific reaction to change physiological state of cells, which can be used for the detection of hazards in food.

In view of this, we focused on the characteristics and performance of electrochemical cell sensors and their applications in the detection of common food hazards. In addition, we mainly studied the surface modification of electrodes, commonly used nanomaterials, cell immobilization and 3D culture. We also discussed the limitations of its commercialization, and provided prospects for the further development of cell-based electrochemical sensors.

## 2. Cell-Based Electrochemical Sensors

### 2.1. Characteristics of Cell-Based Electrochemical Sensors

**Figure 1:**
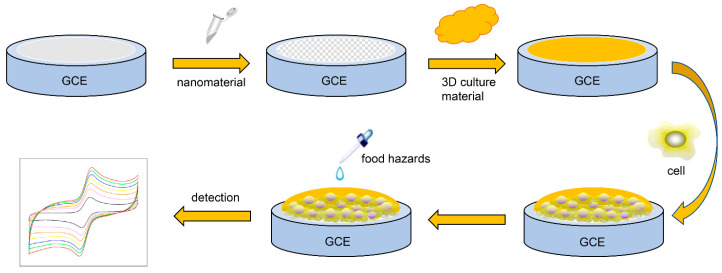
Basic principle of cell-based electrochemical sensors.

A living cell can be properly described as an electrochemical kinetic system. Due to various oxidation–reduction reactions and changes in ionic composition and concentration in biological processes, cell life activities are accompanied by electron generation and charge transfer [13]. Electrochemical methods can be used to reflect the change in cell function and the growth and development of cells. In this case, biochemical parameters such as the concentration of inorganic ions (H^+^, K^+^, Na^+^, Ca^2+^, Cl^−^, etc.), morphological changes, membrane potential and redox potential can be detected by electrochemistry. Figure 1 shows the basic principle of cell-based electrochemical sensors.

Electrochemical impedance spectroscopy (EIS) is the most commonly used technique in the application of cell-based electrochemical sensors to detect usual hazards in food. EIS is an electrochemical technique that can monitor cell adhesion, diffusion and movement in real time [14]. When the cells fixed on the electrode contact with the harmful substances in food, the number, morphology or metabolism of the cells adhering to the electrode surface will change, resulting in a change in electrode interface impedance [15].

### 2.2. Electrode Surface Modification

The successful construction of a cell-based electrochemical sensor mainly depends on the type of electrode used. Choosing the right electrode can effectively improve the performance of the sensor. At present, there are four types of electrodes for cell sensors, namely, glassy carbon electrodes, ITO electrodes, screen-printed electrodes and flexible electrodes. Glassy carbon electrodes are the most basic electrode in electrochemical research, but with the development of cell sensors, the shortcomings of glassy carbon electrodes gradually appear. The shape of the traditional glassy carbon electrode is rod-shaped, and the central position is a conductive carbon rod. Because the conductive interface is vertical and downward when the rod electrode is used for detection, the cells fixed on the electrode easily fall off, and the biological activity of the cells is difficult to maintain; therefore, the electrical conductivity of the sensor will be affected. Moreover, the conductive interface area of the glassy carbon electrode is relatively small, and the response signal is relatively low compared with other electrodes. ITO electrodes are fabricated by magnetron sputtering on a base of sodium calcium or silicon boron substrate glass. ITO electrodes have a large conductive interface, which can effectively improve the response signal, but there are also some problems. The modified material on the electrode is difficult to clean, and it is easy to damage the conductive film, resulting in a decline in the conductivity of the electrode. Therefore, ITO electrodes have a shorter service life than other types of electrodes. Flexible electrodes are usually based on flexible materials such as polydimethylsiloxane or polyethylene terephthalate, on which conductive materials and bioactive materials are used to incubate cells for the detection of target substances [16,17]. Screen-printed electrodes are the most commonly used electrodes in cell-based electrochemical sensors. The most common substrate of screen printing electrodes is paper, and then, the conductive ink is printed on the paper surface by wax printing and screen printing [18]. As the main component of paper is plant fiber, it has porosity, which can provide more anchor points for cell immobilization. At the same time, after modifying some nanomaterials and hydrogels, cells have a better physiological status on the electrodes, which greatly improves the sensor’s ability. In addition, paper-based electrodes are simpler and more low cost, which greatly promotes the development of cell-based electrochemical sensors.

In the process of construction of cell-based electrochemical sensors, adopting different methods to improve the response strength of cell-based sensors is a crucial consideration because certain resistance of a cell will weaken the electrical signal. With the rapid development of biosensors, more and more nanomaterials are applied to biosensors, which also provides some ideas for improving the performance of cell sensors. For instance, some functional nanomaterials such as graphene, carbon nanotubes and gold nanoparticles have been applied to cell-based sensors [19,20,21,22]. These functional nanomaterials can not only improve the response signal, but also provide good compatibility, so that cells can be fixed on the electrode more stably.

In recent years, carbon nanotubes (CNTs) have been widely studied and applied in the field of cell-based electrochemical sensors. According to the number of walls, carbon nanotubes can be divided into single-walled carbon nanotubes (SWCNTs) and multiwalled carbon nanotubes (MWCNTs) [23]. Recent studies have illustrated that CNTs can maintain and promote the electrical activity of neurons in cultured cell networks. Cellot et al. [24] reported a mechanism explaining the effect of CNTs on the collective electrical activity of neural networks. Selhuber-Unkel et al. [25] studied the growth of cells in the network structure of MWCNTs. Compared with SWCNTs, MWCNTs have attracted extensive research interest due to their better electronic, photonic and mechanical properties [26,27,28]. At the same time, some studies have demonstrated that metal nanoparticles (such as gold, platinum and silver) can be adsorbed on the surface of MWCNTs, thus greatly improving the performance of the sensor [29]. Gu et al. [30] decorated platinum nanoparticles on the surface of MWCNTs modified electrode, which greatly improved the conductivity of the electrode and the electron transfer rate, and constructed a sensor with better analytical performance.

Graphene is a two-dimensional nanomaterial with single atom thickness. Due to its excellent electrical conductivity, graphene has shown great prospects in the development of various sensitive cell-based electrochemical sensors [31,32]. One research study reported that intelligent functional graphene films covalently bound with RGD peptides can enhance cell adhesion and growth, and thus, real-time detection of nitric oxide released by adherent human cells stimulated by various drugs. In this study, RGD peptides were covalently bound to pyrene butyric acid-functionalized graphene films to construct biomimetic graphene-based films, which promoted cell attachment and growth [21]. Jiang et al. developed an electrochemical paper-based sensor for the indirect detection of lipopolysaccharide in the outer wall of Gram-negative bacteria. Nafion/polypyrrole/graphene oxide composites have excellent selectivity, high conductivity and good biocompatibility, which can be enhanced by electrochemical polymerization on the working electrode. Mouse macrophage cells encapsulated in alginate hydrogel were used as biological cognitive elements to immobilize Nafion/polypyrrole/graphene oxide screen-printed carbon electrodes in paper fibers [33].

Noble metals have become one of the most popular nanomaterials for modified electrodes due to their good conductivity and catalysis. Noble metals have become one of the nanomaterials for modified electrodes due to their good conductivity and catalysis; the most commonly used noble metal for sensors is gold nanoparticles. Gold nanoparticles can provide binding sites for the coupling of a variety of molecules (enzymes, antibodies, cells), which can be more stable on the surface of the electrode [34]. Therefore, electrodes modified by gold nanoparticles have good stability. Jiang et al. immobilized cysteine on electrodes by combining gold nanoscale and cysteine molecules. Then, cells were adsorbed on the electrode surface by the molecular interaction between cysteine and the hydrogel coated with the cells, and an electrochemical cell sensor with excellent performance was constructed [35]. Due to the good catalytic performance of gold nanoparticles for reactive oxygen species, some researchers have realized the detection of hydrogen peroxide and nitric oxide released by cells by using the characteristics of gold nanoparticles [36,37].

### 2.3. Immobilization and 3D Culture of Cells on Electrodes

Cell-based sensors can be mainly divided into animal, bacterial, and yeast cell-based sensors and bacterial and yeast combination cell-based sensors. The most widely used sensors are animal cell-based sensors and bacterial cell-based sensors based on electrochemical technology to detect food hazards. Cells that are often used in sensors are shown in the table that follows.

**Figure 2:**
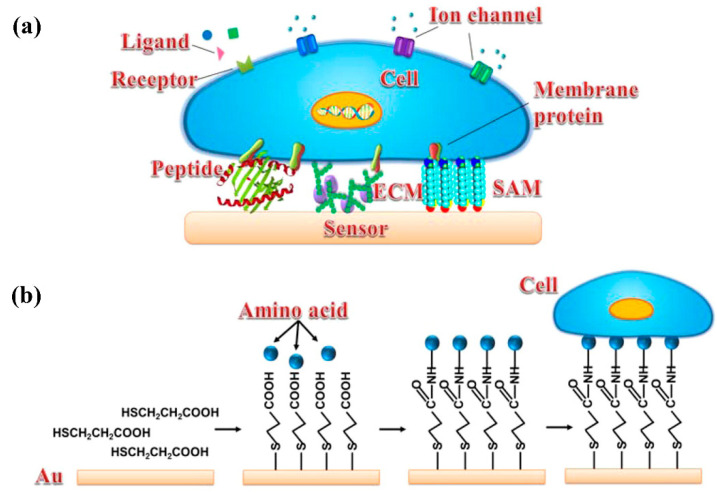
Different chemical modifications of a sensor surface for improving cell immobilization efficiency. (**a**) Schematic diagram of a cell coupled with a sensor surface by different surface modifications, including peptide, ECM, and SAM. (**b**) Schematic of the chemical process of cells immobilized on a gold surface via SAM [38]. Copyright © 2014, American Chemical Society.

The success or failure of a cell-based electrochemical sensor depends on the stability of cell immobilization on the electrode surface. In the current literature, the most commonly used method is to form a uniform adhesion layer on the surface of the electrode, so as to ensure that the cells can be stably fixed on the surface of the electrode. Therefore, the key of cell sensing analysis is to modify the sensing interface by adhesion factors, so that it can specifically adhere to cells without affecting cell function [38]. Cell immobilization materials for cell-based electrochemical sensors are listed in Table 1. Extracellular matrix (ECM) components, peptides, self-assembled monolayer (SAM) and nanomaterials have been used to improve cell immobilization efficiency and performance, as shown in Figure 2.

The cells fixed on the electrode surface are the core part of the whole cell sensor, whose status directly determines the performance of the sensor. So far, most of the sensors for food hazard detection have employed traditional two-dimensional (2D) monolayer cell cultures. Although using 2D cell cultures has proven to be a valuable cell research method with a long history, its limitations are increasingly being recognized. Since almost all cells are surrounded by other cells and extracellular matrix in three dimensions in vivo, the natural three-dimensional (3D) environment of cells is not fully considered in 2D cell cultures. Because the monolayer formed in 2D cultures is different from the multilayer formed in 3D cultures, the inhibition of cell-to-cell contact may hinder the original morphological and functional characteristics of cells. Conversely, 3D cell models can simulate cell conditions in vivo because they have 3D scaffolds that support cell growth and cell functions, including morphogenesis, cell metabolism and intercellular interactions [45]. Therefore, 3D cell culture has become an important technology in the construction of cell sensors.

3D culture mainly depends on the interaction between cells and extracellular matrix substrate. The extracellular matrix is an important material for 3D cell culture, which can act as a scaffold. Recently, a variety of 3D cell scaffolds have emerged. Different types of scaffolds can be used derived from the conditions and expected goals [46]. The development of 3D cell culture technology provides more insight into 3D cell culture scaffolds to simulate the environment of cells in vivo [47]. Wang et al. [48] developed microsphere porous alginate beads (PABs), which can be connected to porous alginate structures by the emulsion of a two-phase aqueous phase (ATPS). The emulsion of two kinds of biocompatible implants of cell/dextran and alginate (Alg)/polyethylene glycol (PEG) is stabilized by mPEG-BSA particles to form ATPS emulsion. Moreover, the pore size of PABs can be adjusted by changing the emulsifying frequency and the volume ratio of the emulsion to the PEG-Alg solution. In addition, due to the good biocompatibility of ATPS, cells can be directly wrapped in interconnected pores. Compared with the cells coated with general alginate beads, HeLa and human hepatoma cells coated with ATPS have stronger cell activity (95%), proliferation and function enhancement. Sun et al. [35] used collagen as a scaffold for cell culture and fixed it on the electrode to detect allergens in shrimp. Jiang et al. [49] designed a paper-based sensor for detecting casein, a common allergen in milk. In their research, cells were coated with gelatin methacryloyl hydrogen and fixed on the working electrode. At the same time, the paper itself had a certain spatial structure so that the growth environment of cells was closer to nature. On the strength of certain spatial structure and low-cost characteristics, paper-based cell sensors have gradually become a research hotspot [50].

## 3. Application of Cell-Based Electrochemical Sensor in Detection of Hazardous Substances in Food

In recent years, food safety has become a key public health problem. Common hazards in food lead to too many safety accidents. At present, common food hazards mainly include some allergens, bacteria and mycotoxins, antibiotics, pesticide residues and some heavy metals. The toxicity of these hazards is also different. After some hazards are ingested by the human body, they may cause fever, allergy, shock or even death. Therefore, the early detection of food hazards becomes very vital. In the past two decades, food safety monitoring technology has made significant progress, and various monitoring methods, including electrochemical methods, have been developed and applied. This section mainly summarized the application of cell sensors based on electrochemical methods in the detection of typical food hazards such as allergens, toxins, antibiotics and other common food hazards in recent years.

### 3.1. Food Allergen

**Figure 3:**
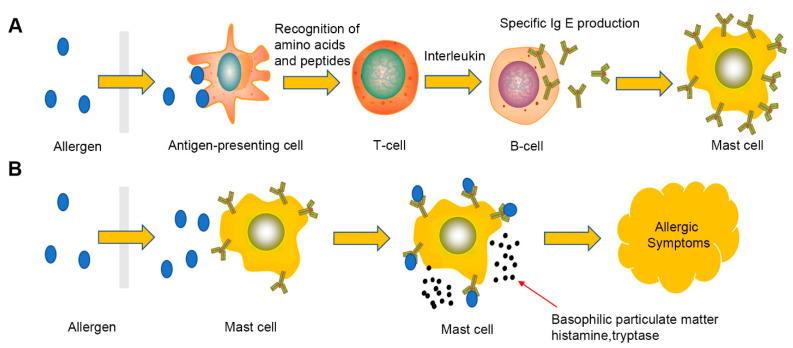
Food allergy mechanism. Food allergy results from an overblown reaction of the immune system to a food allergen. Two steps are necessary for this occurrence: an initial phase of sensitization to a specific antigen (**A**) and the elicitation of an allergic reaction after a second exposure to the same antigen (**B**).

With the development of society, food allergies are receiving considerable attention as a food safety problem. Food allergies are mainly caused by allergen-specific immunoglobulin E (IgE)-mediated immune hypersensitivity, usually for some food proteins, glycoproteins (antigens), cell-mediated (non-IgE) or mixed IgE/cell-mediated. In IgE-mediated food allergies (also known as “real” food allergies), the antigen is recognized by allergen-specific immune cells and causes an immediate allergic reaction after a sensitization stage. In this process, IgE antibodies bind to the surface of effector cells, such as mast cells in tissues or basophils in blood. When the same food allergen comes into contact again, the allergen will bind to mast cells or basophil-bound IgE and crosslink at least two IgE antibodies, which leads to the destruction of the cell membrane and the release of secretions from mast cells and basophil granules such as histamine, neutral protease, and proteoglycan contained in the medium, and triggers classical allergic symptoms. The main mechanism of allergic symptoms is shown in Figure 3.

It is well known that the strategy of building a cell-based electrochemical sensor has become the mainstream method to detect allergens in food. This section listed some typical examples of different allergies, which are exhibited in detail in Table 2. In recent studies, mast cells were most commonly used as sensitive elements of cell-based electrochemical sensors. Mast cells are a kind of basophilic cell, which are widely distributed in mammalian epithelial and connective tissues. The cells are round or oval, and the cytoplasm contains uniform and evenly distributed granular materials [51]. Mast cell anaphylaxis is mediated by specific IgE antibodies, which is needed to activate mast cells before cell immobilization, so that allergens can be accurately identified and detected [52]. When mast cells come into contact with allergens, the cells will degranulate, so we can detect food allergens by monitoring the impedance signal of mast cells. Sun et al. [35] have developed an electrochemical biosensor based on mast cells to quantify tropomyosin and evaluate its IgE-mediated hypersensitivity. In this work, the mast cells of rat basophil leukemia (RBL-2H3) were used. The researchers first dispersed the cells in collagen, and then, used the interaction between gold nanoparticles, L-cysteine and collagen to make the cells stably fixed on the electrode surface, which greatly improved the performance of the sensor. The mast cells were sensitized by nitrophenol bovine serum albumin (DNP-BSA). Then, a mast cell-based biosensor was applied to the quantitative detection of shrimp allergens sensitized by shrimp tropomyosin IgE. The results of EIS showed that the detection limit was 0.15 μg/mL in the range of 0.5–0.25 μg/mL. Jiang et al. [49] designed a paper-based sensor based on mast cells and developed a sensor that can sensitively detect the major allergenic casein in milk. In this study, RBL-2H3 cells were used as sensitive elements, and graphene (GN)/carbon nanofiber (CN)/GelMA was used as a modified material to improve electrical conductivity. At the same time, the paper is used as the working electrode to provide more anchor points for cell 3D growth on the electrode. This greatly improves the stability of the sensor. The results showed that the linear range of casein concentration was from 1 × 10^−7^ g/mL to 1 × 10^−6^ g/mL, and the detection limit was 3.2 × 10^−8^ g/mL. Some other common food allergens, such as the fish allergen parvalbumin and wheat allergens, are gradually detected by cell-based electrochemical sensors [53,54].

### 3.2. Toxins in Food

Toxins in food have also become an important factor affecting food safety. The main sources of these toxins are the toxins existing in food itself, and the toxins produced by some microorganisms in the process of food production and processing due to improper operation. In the past few years, safety incidents caused by food toxins were mainly caused by some fungal toxins. Some typical toxins are mainly produced by Aspergillus, Penicillium or Fusarium, which can cause a series of toxic reactions when ingested above a certain concentration [56]. Therefore, the establishment of a series of simple and effective detection methods for the detection of common toxins in food has become a hot spot. This section listed some typical examples for different toxins, which are exhibited in detail in Table 3.

**Figure 4:**
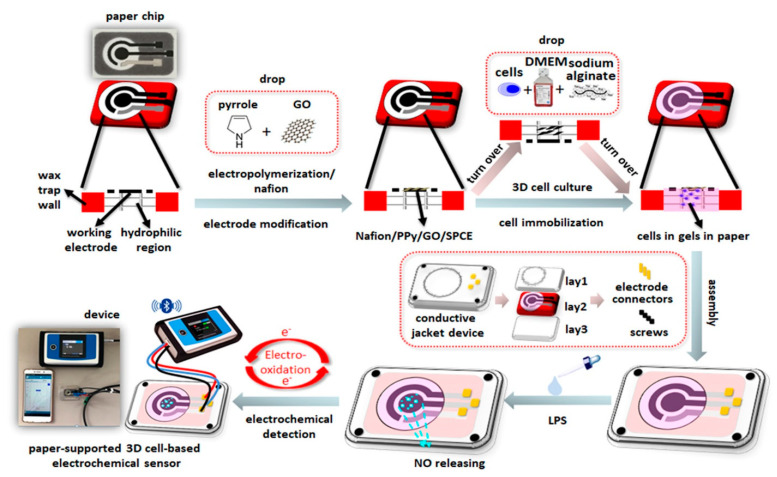
Schematic illustration of the construction and working principle of the miniaturized paper-supported 3D cell-based electrochemical sensor [33]. Copyright © 2020, American Chemical Society.

It is worth noting that cell-based electrochemical sensors have aroused extensive research interest. Gu et al. [20] constructed an electrochemical cell-based sensor to study the cytotoxicity of deoxynivalenol (DON) and zearalenone (ZEN). BEL-7402 cells with good activity and collagen were selected as the recognition elements and scaffold to maintain the cell activity of the sensor, respectively. The cells adhered to the electrode through high affinity between the folate receptor and folate selectivity. EIS was established to evaluate the single and combined toxicity of DON and ZEN. Xia et al. [40] have developed an electrochemical cell biosensor to detect the single or combined toxicity of DON, ZEN and Aflatoxin B_1_ (AFB_1_) to Hep G2 cells. In this research, cells interacted with laminin to form a close cell electrode contact and collagen was used to maintain cell adhesion and viability. EIS was used to evaluate the toxicity of mycotoxin. Jiang et al. [57,58] developed an electrochemical sensor based on mast cells to evaluate the *N*-3-oxododecanoyl homoserine lactone signal molecules of spoilage bacteria populations in freshwater fish. The researchers used alginate/graphene oxide hydrogel-encapsulated mast cells RBL-2H3 and fixed them in screen-printed carbon electrode, and then, dripped the *Pseudomonas aeruginosa* quorum-sensing molecule *N*−3-oxododecanoyl homoserine lactone onto the electrode to record the effect on cell impedance signal. The results show that the sensor has a good linear relationship in the range of 0.1–1 μmol/mL. Therefore, the biosensor can be used as a rapid and accurate detection method, which provides a new way for the real-time monitoring of spoilage bacteria in freshwater fish production. Wang et al. [59] designed an impedance cell sensor using neuroblastoma cells as sensing cells to detect paralytic shellfish-poisoning toxins. The results show that the detection limit of the biosensor is as low as 0.03 ng/mL. The successful establishment of the cell-based electrochemical sensor provides a good method for screening paralytic shellfish poisoning toxins. Ponsonnet et al. [60] designed an impedance cell sensor to detect lipopolysaccharide. Jiang et al. established a paper-based cell sensor for rapid and accurate detection of lipopolysaccharides. The specific experimental steps are shown in Figure 4. In this work, the researchers printed conductive graphite on paper by the screen-printing method to prepare a paper-based cell sensor. The mixture consisting of cell, cell culture medium and alginate hydrogel was dropped onto the electrode, so that cells immobilized on the electrode had a good physiological state. Compared with Ponsonnet’s design [60], the cells can have a better physiological state to detect lipopolysaccharide. The sensor has a good detection effect on lipopolysaccharides; the detection limit of the sensor is 3.5 × 10^−3^ ng/mL and the linear detection range is 1 × 10^−2^ to 3 ng/mL [33].

### 3.3. Other Common Food Hazards

In addition to the common allergens and toxins, there are a number of other hazards such as antibiotics, agricultural and veterinary drug residues, toxic compounds and heavy metals in our diet, which also pose a certain threat to food safety [62,63]. Therefore, cell-based electrochemical biosensors were also chosen to detect these hazards. This section lists some typical examples of the detection of common antibiotics, agricultural and veterinary drug residues, toxic compounds and heavy metal ions in Table 4.

Satpati et al. [64] studied the effect of the antibiotic ciprofloxacin (CIP) on *E. coli*. In this experiment, *E. coli*. was added to the test solution and its volt-ampere peak potential and current were detected by DPV to obtain the interaction pattern between the *E. coli*. and antibiotic CIP. The results showed that *E. coli* gradually cleaved with the increase in CIP concentration. This work provides a potential method to evaluate the toxicity of common antibiotics in food. Mittal et al. [65] used BSA and glutaraldehyde to crosslink and immobilize whole chlorella cells on the platinum surface, and prepared a cell-based electrochemical sensor for detecting heavy metal ions. The principle of the experiment was that alkaline phosphatase in the cell membrane will form a stable metalloenzyme complex with heavy metal ions, thereby reducing the activity of the enzyme. When the activity of the enzyme decreases, it in turn causes the concentration of added phosphate substrate to decrease. This has led to a decrease in the production of p-nitrophenol. When the electroactive nitrophenol was oxidized on the platinum electrode, it caused the current to change, so the detection of heavy metal ions could be realized. Wang et al. [66] constructed a plant cell-based biosensor based on the principle of complexation between alkaline phosphatase and metal ions to monitor the effects of cadmium or lead on plant cells. In this work, the researchers first modified L-cysteine on glassy carbon electrodes, and then, modified anti-IgG-Au antibodies. Then, the cells incubated in vitronectin-like proteins were dropped onto the electrode to explore the damage of heavy metal ions to cells according to the change in impedance. The experimental scheme provides a reference method for the detection of heavy metal ions in food. Yang et al. [67] developed a new type of biosensor using the electrochemical activity of *S. oneidensis* MR-1 cells as a toxicity indicator and 3,5-dichlorophenol as a model toxic compound. In this work, the construction method of this cell sensor is different from other electrochemical cell sensors. The sensor uses a carbon cloth (1 cm × 2 cm) as the working electrode. Then, the electrode was put into the suspension of *S. oneidensis* MR-1 cells for 3,5-dichlorophenol detection. The half maximum inhibitory concentration of 3,5-dichlorophenol measured by the biosensor was about 14.5 mg/L. Thus, this study provides an accurate reference method for evaluating water toxicity.

## 4. Limitations of Cell-Based Electrochemical Sensors

Although cell-based electrochemical sensors have demonstrated great progress and have shown broad application prospects in the field of food, they still face some problems that affect their development [71]. Some of these major problems are further discussed.

The specificity of cell-based electrochemical sensors is a major problem in the detection and analysis of common hazards in food. Due to the complexity of the sample matrix, cell damage may be the result of multiple factors. Therefore, it is difficult to detect specific analytes in complex matrixes only depending on the characteristics of cell damage. At present, researchers have taken some measures to ensure the specificity of cell-based electrochemical sensors. Jiang et al. [35] used the mechanism (see Figure 3) of allergy to realize the specific detection of tropomyosin in shrimp. In order to enable mast cells to specifically recognize the target allergen antigen, the researchers first used tropomyosin antibody IgE to incubate mast cells at 37 °C for 30 min. By co-incubation, IgE antibody was combined with the specific mast cell surface receptor FcεRI to activate the mast cells and enable them to recognize Tropomyosin quickly and accurately [72]. The establishment of this method provides a good idea for the determination of hazardous substances in food. Maintaining the stability of electrochemical cell sensors is also one of the challenges. Because cells are fragile, they are easily affected by temperature, pH and other factors. Therefore, maintaining cell activity on the electrode has become an important direction of cell sensor research.

Moreover, there are some limitations in the commercialization of cell-based electrochemical sensors, mainly as follows: (1) When cell sensors are removed from the laboratory environment, cell delivery and preservation techniques can be problematic. The development of cell-based electrochemical sensors is mainly used for on-site monitoring and testing, and living cells have extremely stringent requirements for the environment. (2) Signal separation has always been a bottleneck of the development of biosensors, and cell-based electrochemical sensors are no exception. Living cells can respond to a variety of substances according to their own receptors and ion channels. It is difficult to separate the signal from the target because of a large number of different substances in food which limits the application of cell sensors. The problems of signal separation, analysis, signal drift and reproducibility need to be solved. (3) The cost of cell culture is relatively high. In addition, disposable cell-based electrochemical sensors greatly increase production costs. At present, the price of commercial electrochemical cell sensors is relatively expensive, and the mass production of sensors is still in its infancy. With the development of sensing technology and the increasing demand for cell sensors, the price of cell-based electrochemical sensors will also decrease in the future.

## 5. Conclusions and Future Perspectives

In summary, cell-based electrochemical sensors have become a promising alternative to traditional technologies over the past few decades. As a powerful piece of analytical equipment, sensors play an important role in food quality and safety, proving fast, economical, high-sensitivity and specific measurements. With the development of microelectronic processing technology and cell culture technology, cell-based electrochemical sensors based on living cells have been developed rapidly. They have shown broad application prospects in the field of food, including the fermentation industry, food safety detection, food sensory bionics (biological nose, biological tongue), shelf-life assessment, food factory environmental monitoring, and so on. Cells provide a series of natural receptors, in which enzymes, receptors, channels and other signaling proteins can be the sites of action of analytes. The unique signal sequence of electrochemical cell sensors has different sensitivity to different kinds of stimuli, which can quantitatively measure and analyze information—that is, determine the existence and concentration of certain substances. Compared with other sensors, it can more realistically reflect the impact of external stimuli.

Cell-based electrochemical sensors have shown great vitality and attractive development prospects in the field of food, and constantly stimulate the research enthusiasm of food scientists and biologists. How to further develop cell immobilization and manipulation methods is of far-reaching significance for cell proliferation and differentiation, detection and analysis. Research at the single-cell level can obtain more accurate and comprehensive information reflecting the physiological state and process of cells. Cell function analysis is becoming more and more important for cell-based electrochemical sensors. Only when cell function is studied clearly can it be more conducive to the development of cell-based electrochemical sensors. In the future, the development direction of electrochemical cell sensors will be to use living tissues, such as biological tissues and cells, as sensitive elements. Combined with microelectronic processing technology, integrated chips are developed to provide an environment suitable for cell survival and growth, and the sensor can be applied to on-site monitoring and analysis. Cell-based electrochemical sensors will become an indispensable tool for food safety, environmental monitoring, drug screening and national defense in the future.

## Figures and Tables

**Table 1:** Cell immobilization materials in electrochemical cell-based sensors.

Immobilization Materials	Electrode Interface	Cell Type	Ref
Collagen	Gold electrode	Mast cell	[35]
Laminin	Screen-printed electrode	Mouse tongue isolated taste bud (MTITB) cells and human embryonic kidney 293 cell lines (HEK293)	[39]
Laminin	Screen-printed electrode	Hep G2 cells	[40]
RGD peptide	Graphene film	Human umbilical vein endothelial cells	[21]
RGD peptide	ITO electrode	Human lung cancer cell A549	[41]
MAST peptide	Gold electrode	Human umbilical vein endothelial cells	[42]
lysine–arginine–glycine–aspartic acid peptide	Gold electrode	Mouse fibroblast cells	[43]
Alkanethiols and polymeric poly-L-lysine-grafted-poly (ethylene glycol)	Gold–silicate interfaces	MCF-7 cells	[44]

**Table 2:** Application of electrochemical cell-based sensor in detection of food allergens.

Cell Types	Analyst	Food	Methods	Performance	Ref.
RBL-2H3 mast cell (RBL-2H3)	tropomyosin	shrimp	EIS	Linear range: 0.5–0.25 μg/mL Detection limit: 0.15 μg/mL	[35]
RBL-2H3	casein	milk	differential pulse voltammetry (DPV)	Linear range: 1 × 10^−^^7^–1 × 10^−6^ g/mL Detection limit: 3.2 × 10^−8^ g/mL	[49]
RBL-2H3	tropomyosin parvalbumin	shrimp fish	EIS	Detection limit: 0.03 μg/mL Detection limit: 0.16 ng/mL	
					[53]
RBL-2H3, ANA-1macrophages	dinitrophenylated bovine serum albumin	-	EIS	Cell co-culture model Detection limit: 10^−1^ ng/mL	[55]
RBL-1	wheat protein	wheat	cyclic voltammetry (CV)	Linear range: 0.01–0.5 μg/mL	[54]

**Table 3:** Application of electrochemical cell-based sensors in detection of food toxins.

Cell Types	Analyst	Food	Methods	Performance	Ref.
RBL-2H3	N-acyl-homoserine-lactones	fish	EIS	Linear range: 0.1–1 μmol/L Detection limit: 0.034 μmol/L	[57]
HeLa & HepG2 cell	okadaic acid	shellfish	Electrical cell-substrate impedance sensing (ECIS)	Detection limit: 10.2 μg/L	[61]
Neuroblastoma cell	saxitoxin, ouabain, veratridine	shellfish	EIS	Detection limit: 0.03 ng/mL	[59]
BEL-7402 cell	DON, ZEN	-	EIS	Linear range: 0.1–20 μg/Ml, 0.1–50 μg/mL Detection limit: 0.03 μg/mL, 0.05 μg/mL	[20]
Hep G2 cell	DON, ZEN, AFB_1_	-	EIS	Linear range: 0.01–20, 0.1–50 and 0.1–3.5 μg/mL	[40]
Raw264.7 macrophage cells	lipopolysaccharide	juice	DPV	Linear range: 1 × 10^−2^–3 nmol/L Detection limit: 3.5 × 10^−3^ ng/mL	[33]

**Table 4:** Application of cell-based electrochemical sensors in the detection of antibiotics, agricultural and veterinary drug residues, toxic compounds and heavy metals in food.

Cell Types	Analyst	Food	Methods	Performance	Ref.
*S. oneidensis* MR-1	3,5-dichlorophenol	water	Amperometric i–t curve	half maximal inhibitory concentration: 14.5 mg/L	[67]
microalgae *Chlorella* sp.	Zn^2+^	water	DPV	Linear range: 10^−12^–10^−10^ mol/L Detection limit: 10^−11^ mol/L	[65]
plant cell (protoplasts)	Cd^2+^, Pb^2+^	soybean	EIS	Linear range: 45–210 μmol/L, 120–360 μmol/L Detection limit: 18.5 nmol/L, 25.6 nmol/L	[66]
*E. coli* cell	ciprofloxacin	-	DPV	the binding constant of E. coli membrane protein F and CIP log K_f_ = 12.1	[64]
BPAECs	lindane	drinking water	ECIS	Detection limit: 0.1 mmol/L	[68]
*Arthrospira platensis* cell	Cd^2+^, Hg^+^	water	Lock-in amplifier method	Linear range: 10^−20^–10^−6^ mol/L Detection limit: 10^−20^ mol/L	[69]
*P. aeruginosa* cell	cephalosporin group of antibiotics	-	-	Linear range: 0.1–11 mmol/L	[70]

## References

[1] Borda D., Mihalache O.A., Dumitraşcu L., Gafițianu D., Nicolau A.I. (2021). Romanian consumers’ food safety knowledge, awareness on certified labelled food and trust in information sources. Food Control.

[2] Todd E.C.D. (2014). Foodborne Diseases: Overview of Biological Hazards and Foodborne Diseases. Encycl. Food Saf..

[3] Ma Y., Ding S., Fei Y., Liu G., Jang H., Fang J. (2019). Antimicrobial activity of anthocyanins and catechins against foodborne pathogens Escherichia coli and Salmonella. Food Control.

[4] Rivera D., Toledo V., Reyes-Jara A., Navarrete P., Tamplin M., Kimura B., Wiedmann M., Silva P., Moreno Switt A.I. (2018). Approaches to empower the implementation of new tools to detect and prevent foodborne pathogens in food processing. Food Microbiol..

[5] Narwal V., Deswal R., Batra B., Kalra V., Hooda R., Sharma M., Rana J.S. (2019). Cholesterol biosensors: A review. Steroids.

[6] Gui Q., Lawson T., Shan S., Yan L., Liu Y. (2017). The Application of Whole Cell-Based Biosensors for Use in Environmental Analysis and in Medical Diagnostics. Sensors.

[7] Xu G., Wu Y., Li R., Wang P., Yan W., Zheng X. (2002). Cell-based biosensors:Towards the development of cellular monitoring. Chin. Sci. Bull..

[8] Banerjee P., Kintzios S., Prabhakarpandian B. (2013). Biotoxin detection using cell-based sensors. Toxins.

[9] Jia X., Tan L., Zhou Y., Jiang X., Xie Q., Tang H., Yao S. (2009). Magnetic immobilization and electrochemical detection of leukemia K562 cells. Electrochem. Commun..

[10] Weng J., Zhang Z., Sun L., Wang J.A. (2011). High sensitive detection of cancer cell with a folic acid-based boron-doped diamond electrode using an AC impedimetric approach. Biosens. Bioelectron..

[11] Zhong X., Bai H.-J., Xu J.-J., Chen H.-Y., Zhu Y.-H. (2010). A Reusable Interface Constructed by 3-Aminophenylboronic Acid-Functionalized Multiwalled Carbon Nanotubes for Cell Capture, Release, and Cytosensing. Adv. Funct. Mater..

[12] Jing-Jing Z., Fang-Fang C., Ting-Ting Z., Jun-Jie Z. (2010). Design and implementation of electrochemical cytosensor for evaluation of cell surface carbohydrate and glycoprotein. Anal. Chem..

[13] Nonner W. (2000). Electrodiffusion in ionic channels of biological membranes. J. Mol. Liqs..

[14] Wegener J., Keese C.R., Giaever I. (2000). Electric cell-substrate impedance sensing (ECIS) as a noninvasive means to monitor the kinetics of cell spreading to artificial surfaces. Exp. Cell Res..

[15] Zhang X., Wang W., Nordin A.N., Li F., Jang S., Voiculescu I. (2017). The influence of the electrode dimension on the detection sensitivity of electric cell–substrate impedance sensing (ECIS) and its mathematical modeling. Sens. Actuators B Chem..

[16] Zhao X., Wang K., Li B., Wang C., Ding Y., Li C., Mao L., Lin Y. (2018). Fabrication of a Flexible and Stretchable Nanostructured Gold Electrode Using a Facile Ultraviolet-Irradiation Approach for the Detection of Nitric Oxide Released from Cells. Anal. Chem..

[17] Lin Y., Bariya M., Nyein H.Y.Y., Kivimäki L., Uusitalo S., Jansson E., Ji W., Yuan Z., Happonen T., Liedert C. (2019). Porous Enzymatic Membrane for Nanotextured Glucose Sweat Sensors with High Stability toward Reliable Noninvasive Health Monitoring. Adv. Funct. Mater..

[18] Kokkinos C., Economou A., Giokas D. (2018). Paper-based device with a sputtered tin-film electrode for the voltammetric determination of Cd(II) and Zn(II). Sens. Actuators B: Chem.

[19] Justino C.I.L., Gomes A.R., Freitas A.C., Duarte A.C., Rocha-Santos T.A.P. (2017). Graphene based sensors and biosensors. TrAC Trends Anal. Chem..

[20] Gu W., Zhu P., Jiang D., He X., Li Y., Ji J., Zhang L., Sun Y., Sun X. (2015). A novel and simple cell-based electrochemical impedance biosensor for evaluating the combined toxicity of DON and ZEN. Biosens. Bioelectron..

[21] Xian G.C., Rui N.S., Yun K.S., Xinting Z., Peng C., Ming L.C. (2012). RGD-peptide functionalized graphene biomimetic live-cell sensor for real-time detection of nitric oxide molecules. ACS Nano.

[22] Viswanathan S., Radecka H., Radecki J. (2009). Electrochemical biosensors for food analysis. Mon. Chem. Chem. Mon..

[23] Tilmaciu C.M., Morris M.C. (2015). Carbon nanotube biosensors. Front. Chem..

[24] Cellot G., Cilia E., Cipollone S., Rancic V., Sucapane A., Giordani S., Gambazzi L., Markram H., Grandolfo M., Scaini D. (2009). Carbon nanotubes might improve neuronal performance by favouring electrical shortcuts. Nat. Nanotechnol..

[25] Taale M., Schutt F., Zheng K., Mishra Y.K., Boccaccini A.R., Adelung R., Selhuber-Unkel C. (2018). Bioactive Carbon-Based Hybrid 3D Scaffolds for Osteoblast Growth. ACS Appl. Mater. Interfaces.

[26] Li H., Chen R., Ali M., Lee H., Ko M.J. (2020). In Situ Grown MWCNTs/MXenes Nanocomposites on Carbon Cloth for High-Performance Flexible Supercapacitors. Adv. Funct. Mater..

[27] Yao L., Teng J., Zhu M., Zheng L., Zhong Y., Liu G., Xue F., Chen W. (2016). MWCNTs based high sensitive lateral flow strip biosensor for rapid determination of aqueous mercury ions. Biosens. Bioelectron..

[28] Zhang R., Ying C., Gao H., Liu Q., Fu X., Hu S. (2019). Highly flexible strain sensors based on polydimethylsiloxane/carbon nanotubes (CNTs) prepared by a swelling/permeating method and enhanced sensitivity by CNTs surface modification. Compos. Sci. Technol..

[29] Guo J.W., Zhang B., Hou Y., Yang S., Yang X.H., Yang H.G. (2013). A sulfur-assisted strategy to decorate MWCNTs with highly dispersed Pt nanoparticles for counter electrode in dye-sensitized solar cells. J. Mater. Chem. A.

[30] Li J., Huang X., Shi W., Jiang M., Tian L., Su M., Wu J., Liu Q., Yu C., Gu H. (2021). Pt nanoparticle decorated carbon nanotubes nanocomposite based sensing platform for the monitoring of cell-secreted dopamine. Sens. Actuators B Chem..

[31] Vlasceanu G.M., Amarandi R.M., Ionita M., Tite T., Iovu H., Pilan L., Burns J.S. (2018). Versatile graphene biosensors for enhancing human cell therapy. Biosens. Bioelectron..

[32] Taniselass S., Arshad M.K.M., Gopinath S.C.B. (2019). Graphene-based electrochemical biosensors for monitoring noncommunicable disease biomarkers. Biosens. Bioelectron..

[33] Jiang H., Yang J., Wan K., Jiang D., Jin C. (2020). Miniaturized Paper-Supported 3D Cell-Based Electrochemical Sensor for Bacterial Lipopolysaccharide Detection. ACS Sens..

[34] Minati L., Antonini V., Torrengo S., Serra M.D., Boustta M., Leclercq X., Migliaresi C., Vert M., Speranza G. (2012). Sustained in vitro release and cell uptake of doxorubicin adsorbed onto gold nanoparticles and covered by a polyelectrolyte complex layer. Int. J. Pharm..

[35] Jiang D., Ji J., An L., Sun X., Zhang Y., Zhang G., Tang L. (2013). Mast cell-based electrochemical biosensor for quantification of the major shrimp allergen Pen a 1 (tropomyosin). Biosens. Bioelectron..

[36] Li J., Xie J., Gao L., Li C.M. (2015). Au nanoparticles-3D graphene hydrogel nanocomposite to boost synergistically in situ detection sensitivity toward cell-released nitric oxide. ACS Appl. Mater. Interfaces.

[37] Fan W.T., Qin Y., Hu X.B., Yan J., Wu W.T., Liu Y.L., Huang W.H. (2020). Stretchable Electrode Based on Au@Pt Nanotube Networks for Real-Time Monitoring of ROS Signaling in Endothelial Mechanotransduction. Anal Chem..

[38] Liu Q., Wu C., Cai H., Hu N., Zhou J., Wang P. (2014). Cell-Based Biosensors and Their Application in Biomedicine. Chem. Rev..

[39] Deng Y., Zheng H., Yi X., Shao C., Xiang B., Wang S., Zhao Z., Zhang X., Hui G. (2019). Paralytic shellfish poisoning toxin detection based on cell-based sensor and non-linear signal processing model. Int. J. Food Prop..

[40] Xia S., Zhu P., Pi F., Zhang Y., Li Y., Wang J., Sun X. (2017). Development of a simple and convenient cell-based electrochemical biosensor for evaluating the individual and combined toxicity of DON, ZEN, and AFB1. Biosens. Bioelectron..

[41] Li Y., Yu C. (2017). RGD peptide doped polypyrrole film as a biomimetic electrode coating for impedimetric sensing of cell proliferation and cytotoxicity. J. Appl. Biomed..

[42] Hitoshi A., Katsumi M., Tetsuya H. (2008). Seamless signal transduction from live cells to an NO sensor via a cell-adhesive sensing matrix. Anal. Chem..

[43] Asphahani F., Thein M., Veiseh O., Edmondson D., Kosai R., Veiseh M., Xu J., Zhang M. (2008). Influence of cell adhesion and spreading on impedance characteristics of cell-based sensors. Biosens. Bioelectron..

[44] Wu Y., Lian J., Goncales V.R., Pardehkhorram R., Tang W., Tilley R.D., Gooding J.J. (2020). Patterned Molecular Films of Alkanethiol and PLL-PEG on Gold-Silicate Interfaces: How to Add Functionalities while Retaining Effective Antifouling. Langmuir.

[45] Foglietta F., Canaparo R., Muccioli G., Terreno E., Serpe L. (2020). Methodological aspects and pharmacological applications of three-dimensional cancer cell cultures and organoids. Life Sci..

[46] Wang X., Dai X., Zhang X., Li X., Xu T., Lan Q. (2018). Enrichment of glioma stem cell-like cells on 3D porous scaffolds composed of different extracellular matrix. Biochem. Biophys. Res. Commun..

[47] Xiao Y., Zhou M., Zhang M., Liu W., Zhou Y., Lang M. (2019). Hepatocyte culture on 3D porous scaffolds of PCL/PMCL. Colloids Surf. B Biointerfaces.

[48] Liu T., Yi S., Liu G., Hao X., Du T., Chen J., Meng T., Li P., Wang Y. (2021). Aqueous two-phase emulsions-templated tailorable porous alginate beads for 3D cell culture. Carbohydr. Polym..

[49] Jiang D., Ge P., Wang L., Jiang H., Yang M., Yuan L., Ge Q., Fang W., Ju X. (2019). A novel electrochemical mast cell-based paper biosensor for the rapid detection of milk allergen casein. Biosens. Bioelectron..

[50] Liang L., Su M., Li L., Lan F., Yang G., Ge S., Yu J., Song X. (2016). Aptamer-based fluorescent and visual biosensor for multiplexed monitoring of cancer cells in microfluidic paper-based analytical devices. Sens. Actuators B Chem..

[51] Roberts I.S.D., Brenchley P.E.C. (2000). Mast cells: The forgotten cells of renal fibrosis. J. Clin. Pathol..

[52] Becker M., Lemmermann N.A., Ebert S., Baars P., Renzaho A., Podlech J., Stassen M., Reddehase M.J. (2015). Mast cells as rapid innate sensors of cytomegalovirus by TLR3/TRIF signaling-dependent and -independent mechanisms. Cell. Mol. Immunol..

[53] Jiang D., Zhu P., Jiang H., Ji J., Sun X., Gu W., Zhang G. (2015). Fluorescent magnetic bead-based mast cell biosensor for electrochemical detection of allergens in foodstuffs. Biosens. Bioelectron..

[54] Nakamura N., Kumazawa S., Matsunaga T. (1995). In vitro electrochemical detection of wheat allergen using rat basophilic leukaemia (RBL-1) cells. J. Appl. Microbiol. Biotechnol..

[55] Jiang H., Jiang D., Zhu P., Pi F., Ji J., Sun C., Sun J., Sun X. (2016). A novel mast cell co-culture microfluidic chip for the electrochemical evaluation of food allergen. Biosens. Bioelectron..

[56] Streit E., Schatzmayr G., Tassis P., Tzika E., Marin D., Taranu I., Tabuc C., Nicolau A., Aprodu I., Puel O. (2012). Current situation of mycotoxin contamination and co-occurrence in animal feed--focus on Europe. Toxins.

[57] Jiang D., Feng D., Jiang H., Yuan L., Yongqi Y., Xu X., Fang W. (2017). Preliminary study on an innovative, simple mast cell-based electrochemical method for detecting foodborne pathogenic bacterial quorum signaling molecules (N-acyl-homoserine-lactones). Biosens. Bioelectron..

[58] Jiang D., Liu Y., Jiang H., Rao S., Fang W., Wu M., Yuan L., Fang W. (2018). A novel screen-printed mast cell-based electrochemical sensor for detecting spoilage bacterial quorum signaling molecules (N-acyl-homoserine-lactones) in freshwater fish. Biosens. Bioelectron..

[59] Zou L., Wu C., Wang Q., Zhou J., Su K., Li H., Hu N., Wang P. (2015). An improved sensitive assay for the detection of PSP toxins with neuroblastoma cell-based impedance biosensor. Biosens. Bioelectron..

[60] Bouafsoun A., Othmane A., Jaffrézic-Renault N., Kerkeni A., Thoumire O., Prigent A.F., Ponsonnet L. (2008). Impedance endothelial cell biosensor for lipopolysaccharide detection. Mater. Sci. Eng. C.

[61] Zou L., Wang Q., Tong M., Li H., Wang J., Hu N., Wang P. (2016). Detection of diarrhetic shellfish poisoning toxins using high-sensitivity human cancer cell-based impedance biosensor. Sens. Actuators B Chem..

[62] McDermott P.F., Zhao S., Wagner D.D., Simjee S., Walker R.D., White D.G. (2002). The food safety perspective of antibiotic resistance. Anim. Biotechnol..

[63] Bird S.B., Sutherland T.D., Gresham C., Oakeshott J., Scott C., Eddleston M. (2008). OpdA, a bacterial organophosphorus hydrolase, prevents lethality in rats after poisoning with highly toxic organophosphorus pesticides. Toxicology.

[64] Ipte P.R., Satpati A.K. (2020). Probing the interaction of ciprofloxacin and E. coli by electrochemistry, spectroscopy and atomic force microscopy. Biophys. Chem..

[65] Singh J., Mittal S.K. (2012). Whole cell based amperometric sensor with relative selectivity for zinc ions. Anal. Methods.

[66] Wang X., Cheng M., Yang Q., Wei H., Xia A., Wang L., Ben Y., Zhou Q., Yang Z., Huang X. (2019). A living plant cell-based biosensor for real-time monitoring invisible damage of plant cells under heavy metal stress. Sci. Total Environ..

[67] Yang Y., Wang Y.Z., Fang Z., Yu Y.Y., Yong Y.C. (2018). Bioelectrochemical biosensor for water toxicity detection: Generation of dual signals for electrochemical assay confirmation. Anal. Bioanal. Chem..

[68] Curtis T.M., Widder M.W., Brennan L.M., Schwager S.J., van der Schalie W.H., Fey J., Salazar N. (2009). A portable cell-based impedance sensor for toxicity testing of drinking water. Lab Chip..

[69] Tekaya N., Saiapina O., Ouada H.B., Lagarde F., Namour P., Ouada H.B., Jaffrezic-Renault N. (2014). Bi-Enzymatic Conductometric Biosensor for Detection of Heavy Metal Ions and Pesticides in Water Samples Based on Enzymatic Inhibition in Arthrospira platensis. J. Environ. Prot..

[70] Kumar S., Kundu S., Pakshirajan K., Dasu V.V. (2008). Cephalosporins determination with a novel microbial biosensor based on permeabilized Pseudomonas aeruginosa whole cells. Appl. Biochem. Biotechnol..

[71] Curulli A. (2021). Electrochemical Biosensors in Food Safety: Challenges and Perspectives. Molecules.

[72] Kim T.E., Park S.W., Cho N.Y., Choi S.Y., Yong T.S., Nahm B.H., Lee S., Noh G. (2002). Quantitative Measurement of Serum Allergen-Specific IgE on Protein Chip. Exp. Mol. Med..

